# Orchestrating Embodied Systems through the Embodied Context Protocol: Motivation, Progress, and Directions

**DOI:** 10.34133/research.1047

**Published:** 2025-12-23

**Authors:** Fuyu Ma, Dong Li, Yizhe Chen, Jiahui Xing, Yu Liu, Dapeng Lan, Jinyan Shao, Zhibo Pang

**Affiliations:** ^1^ Shenyang Institute of Automation, Chinese Academy of Sciences, Shenyang, China.; ^2^ University of Chinese Academy of Sciences, Beijing, China.; ^3^ Shandong Normal University, Jinan, Shandong, China.; ^4^ Shenyang University of Chemical Technology, Shenyang, China.; ^5^ Peking University, Beijing, China.

## Abstract

The emergence of embodied intelligence has brought a fundamental shift to the robotics field, emphasizing the integration of perception, cognition, and control in dynamic physical environments. Although substantial progress has been made in artificial intelligence models, control middleware, and industrial communication protocols within their respective domains, the fragmentation in semantic interaction and task-level coordination still limits the scalability and deployment of embodied intelligence systems. This review synthesizes the current research on system deployment and coordination in embodied intelligence, particularly focusing on the challenges in achieving task-level coordination and semantic interoperability across heterogeneous components. We examine key coordination requirements, such as context semantics, capability declaration, and workflow composition, and highlight the existing gaps in addressing these issues within current systems. In response, we propose the Embodied Context Protocol (ECP) as an emerging solution, designed to bridge these gaps and enhance interoperability across various subsystems. It then presents the design philosophy, interface specification, and execution workflow of ECP, followed by its current implementation progress validated through practical deployments, while also highlighting the future directions and unresolved challenges that will shape its standardization and large-scale adoption. As an interface protocol, ECP aims to evolve into a standardized interoperability ecosystem for embodied intelligence and industrial automation. Realizing this vision will require collaboration across academic and industrial communities to jointly advance the development, adoption, and standardization of ECP.

## Introduction

As humanoid robots begin to leave research labs and enter factories, hospitals, and homes, the global robotics community is witnessing an accelerated shift—from task-specific automation to cognitive, context-aware embodied systems [1,2]. International frontrunners such as Tesla, Boston Dynamics, and Unitree Robotics are investing heavily in building embodied agents [3,4]. At the same time, research communities are actively exploring large-scale policy models, multimodal representation learning, and high-fidelity simulation environments to enable robust decision-making and actuation [5,6]. To support this transition, governments and national laboratories have also initiated efforts to standardize the architectures of next-generation robotic systems, aiming to ensure interoperability, safety, and cross-domain scalability [7–9]. Collectively, these trends indicate that embodied intelligence not only is a cutting-edge research domain but has also become a global technological race.

However, as these systems move toward real-world deployment, coordination bottlenecks at the interface level have become salient. While artificial intelligence (AI) models, control middleware (e.g., Robot Operating System [ROS]), and industrial communication protocols (e.g., Open Platform Communications Unified Architecture [OPC UA]) have each matured individually, they remain fragmented in their ability to communicate semantically and coordinate execution coherently [10–12]. With the increasing modularity and complexity of embodied systems, such fragmentation further exacerbates coordination overhead, weakens cross-module generalization, and limits the scalability of task execution.

To address these coordination issues, we conducted a systematic literature review of existing studies in robotics and embodied intelligence, as well as standardization efforts in related domains, analyzing works from key databases over the past 5 years. We focused on studies related to task coordination, semantic interoperability, and the integration of real-world and simulation systems. Through a systematic analysis, we found that current embodied systems lack a unified interface protocol capable of integrating system components and enabling seamless coordination across heterogeneous modules.

Focusing on this core finding, we conducted a qualitative synthesis of the literature to derive the concrete requirements for a unified interface in embodied systems, including semantic consistency, interoperability, task-level coordination, and simulation-to-real consistency. Grounded in both theoretical studies and real-world deployment experience, these requirements expose a structural gap between AI models, embodied robotic stacks, and industrial automation systems. To meet these needs, we introduce the Embodied Context Protocol (ECP) as the unified interface protocol for embodied systems. It has 4 layers: the Semantic Layer ensures consistent representation of observations, actions, and task context, with explicit units, frames, and timestamps; the Interaction Layer defines a small, closed set of interface verbs and a uniform progress and failure envelope that supports supervision, time-out handling, and recovery; the Adapter Layer normalizes units, frames, and clocks and validates interfaces so that the same call sequence behaves equivalently in simulation and on hardware; and the Workflow Layer composes operations into executable task flows that preserve interoperability across heterogeneous platforms and remain portable when backends change. Together, these layers mitigate schema drift, standardize progress semantics, improve simulation-to-real consistency, and reduce redeployment effort.

The contributions of this review-driven proposal are 3-fold:

• a structured review of embodied intelligence research progress, system orchestration and coordination challenges, and standards in related domains
• a systematic synthesis of key requirements for unified interfaces, derived from both literature and deployment analysis
• the ECP proposal, including its design rationale, interface specification, and workflow semantics, positioned as a standardization candidate informed by the above synthesis and demonstrated through functional implementations

The remainder of this paper is organized as follows: Background and Related Work reviews the evolution of embodied intelligence and analyzes coordination challenges and standardization efforts across related domains. Requirements outlines the core requirements for unified interface protocols in embodied system deployments, based on architectural abstraction and real-world case analysis. Overview of ECP presents the ECP protocol, covering its design rationale, interface specification, execution workflow, and current work in progress as a proposal emerging from the review. Future Directions and Challenges discusses future directions, along with important unresolved challenges that will shape its standardization and large-scale adoption. Finally, Conclusion concludes the review.

## Background and Related Work

This section provides a comprehensive overview of embodied intelligence, its architectural evolution, and the coordination challenges that arise as these systems move toward large-scale deployment. To contextualize these challenges, we conduct a structured review of existing interface paradigms and standardization efforts across AI, industrial automation, and robotics. The review examines how these frameworks address data exchange, capability representation, and coordination semantics and discusses their practical limitations in achieving seamless integration between cognition, control, and physical execution in embodied tasks.

### The rise of embodied intelligence

Embodied intelligence represents a new paradigm in robotics, characterized by the tight integration of perception, cognition, action, and environment interaction [13]. In contrast to conventional AI paradigms, which are predominantly disembodied and confined to static datasets or simulations [14], embodied intelligence emphasizes active and continuous physical engagement, enabling agents to autonomously perceive multimodal sensory inputs, accumulate experiential knowledge, and dynamically adapt through real-time feedback loops with their environment [15].

Recent advancements in embodied intelligence have been accelerated by breakthroughs in multimodal foundation models and large language models (LLMs) [16,17]. These models provide robots with advanced cognitive abilities, allowing seamless translation from human instructions into sophisticated physical actions, particularly in complex industrial tasks requiring high adaptability and context awareness [18]. Embodied agents now leverage structured cognitive–motor architectures, often analogized as a functional triad consisting of an AI-driven cerebrum for high-level decision-making, a cerebellum-like subsystem for fine-grained motor coordination, and sensorimotor interfaces constituting the physical embodiment [2,19,20]. This multimodal architecture is increasingly capable of supporting robust cross-modal interaction and scalable generalization, paving the way for more humanlike and adaptive AI systems across complex environments [21].

However, as embodied agents are deployed in increasingly complex and dynamic environments, there is a growing need for tightly integrated cognitive architectures. A key challenge lies in achieving seamless coordination and interoperability between perception, memory, reasoning, and action components within the agent system [22,23]. Addressing these challenges is critical for the scalability and reliability of embodied intelligent systems, a topic that will be explored in the subsequent section.

### Coordination challenges in embodied systems

**Fig. 1.**
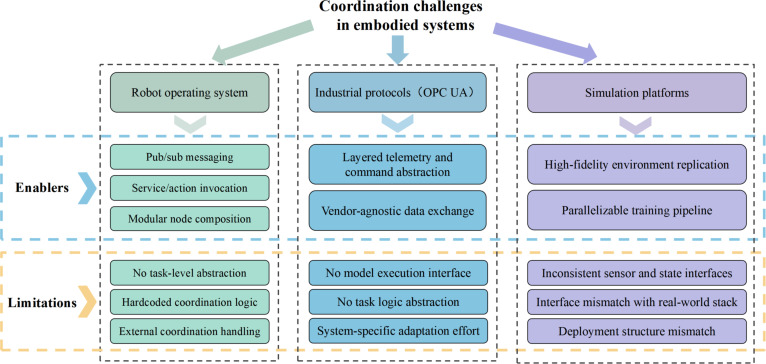
Coordination limitations occur when robot operating systems, industrial protocols, and simulation platforms are applied to embodied tasks. OPC UA, Open Platform Communications Unified Architecture.

As illustrated in Fig. 1, with embodied intelligence moving toward real-world deployment, coordinating perception, planning, and control across diverse subsystems has become a central challenge that existing protocols and tools cannot adequately address.

First, ROS has become a foundational middleware in robotic development due to its flexible publish–subscribe architecture and support for modular design [24]. While ROS 2 represents a substantial evolution from the original ROS framework, it retains the use of predefined communication mechanisms to connect distributed components [25]. Topics, services, and actions serve as message-passing channels in ROS 2, facilitating communication among distributed nodes, but they do not natively support task-level abstraction or orchestration [26]. As robotic systems scale, the lack of native abstractions for intent, contextual reasoning, and execution semantics often forces developers to adopt ad hoc integration strategies—such as hardcoding internode logic or off-loading coordination to external scripts [27]. This results in fragile system architectures that are difficult to extend, debug, or generalize across deployments.

In addition, protocols like OPC UA and MQTT are widely used in industrial automation for structured data exchange across machines and control systems. These protocols provide stable, layered abstractions for telemetry, command, and alarm signals. However, they are agnostic to AI-based model execution and task-level reasoning [28]. As a result, embodied tasks, especially those involving perception-driven control and adaptive decision-making, require developers to embed execution logic atop these protocols in nonstandardized ways [29]. This leads to fragmented control pathways, redundant effort in system adaptation, and heavy reliance on system-specific engineering expertise. As task granularity and cross-module dependencies increase, these implicit coupling patterns become a source of inefficiency and technical debt.

Finally, simulation is indispensable for training, testing, and validating embodied policies. Platforms such as Isaac Sim and Gazebo enable scalable development in robotics by accurately replicating complex static and dynamic environments [30]. However, simulation pipelines are typically constructed in isolation from real-world control stacks, leading to a disconnect in both data representation and interface semantics [31,32]. Models trained in simulation often rely on idealized sensory input formats and control abstractions that are mismatched with those used in deployment. Bridging this gap requires extensive engineering effort to rebind sensors, rewrap outputs, and reinterpret state representations—undermining the scalability of embodied learning pipelines [33]. The lack of consistent structure between simulated and real-world modules hinders reproducibility, increases deployment latency, and reduces overall system robustness.

### Overview of standardization efforts in related domains

As coordination challenges continue to hinder the scalability of embodied systems, it becomes necessary to examine whether existing protocols and standardization efforts in AI, industrial automation, and robotics can offer viable solutions. To this end, we surveyed a set of representative standards and protocols, as summarized in Table 1.

**Table 1.** Overview of representative protocols and standards in AI, industrial automation, and robotics

Protocol/standard	Domain	Standardization scope	Representative mechanism	Status
IEEE 7001-2021 [64]	AI/autonomous systems	Establishes measurable transparency levels for autonomous systems	Transparency levels for stakeholders; System Transparency Assessment; System Transparency Specification	Active standard
IEEE P3526 [65]	AI/cybersecurity	Defines a multilayered LLM-based framework for threat intelligence retrieval	LLM-enabled 4-layer retrieval architecture with modular semantic parsing and query execution	Active PAR
ARIO (All Robots in One) [40]	AI/embodied AI dataset	Defines a unified multimodal data format for embodied agents across robots, tasks, and environments	Hierarchical structure (series–task–episode), timestamped multimodality (RGB, 3D, sound, text, and tactile)	Proposed (academic standard)
MCP (Model Context Protocol) [35,66,67]	AI/LLM interaction	Defines a unified protocol for context, memory, and tool interoperability across AI agents	JSON-RPC interface with prompt.submit, tool.execute, and resource.get; standardized context tokens	Open protocol (nonstandardized)
ISO/IEC 42001:2023 [68]	AI/management systems	Specifies requirements for establishing, implementing, maintaining, and improving an AI management system	Risk management, transparency, accountability, human oversight, and continuous improvement	Active standard
IEEE P2874 [69]	AI/Spatial Web	Defines concepts, architecture, protocols, and governance for the Spatial Web	Hyperspace Modeling Language; Hyperspace Transaction Protocol; Universal Domain Graph	Active PAR
IEEE P2975.2 [70]	Industrial automation/AI	Defines verification and validation requirements for AI models in manufacturing	Addresses model bias, drift, accuracy, and consistency constraints	Active PAR
IEEE P2975.3 [71]	Industrial automation/AI	Describes a software framework for industrial AI, including features and interfaces	Recommendations for AI software frameworks in industrial control systems	Active PAR
IEEE P2976 [72]	Industrial automation/AI	Specifies requirements for AI systems to be recognized as explainable, enhancing clarity and interoperability	Defines mandatory and optional explainability criteria; XML schema for system descriptions	Active PAR
IEC 62541 [73]	Industrial automation/communication	Specifies OPC UA for secure, platform-independent communication in industrial systems	Information modeling; client–server and publish–subscribe communication; robust security features	Active standard
IEEE P3023 [74]	Industrial automation/cloud platform	Defines intelligent interaction systems based on industrial cloud platforms	Specifies functions and relationships of user, demand access, and service layers	Active PAR
IEC 61499 [75]	Industrial automation/distributed control	Provides a reference architecture for distributed control systems using function blocks	Event-driven function blocks with Execution Control Charts, supporting modularity and reusability	Active standard
IEEE 1451.0-2024 [76]	Industrial automation/transducer interface	Defines common functions, communication protocols, and TEDS formats for smart transducers	Network and transducer services, APIs, UUIDs, security, and time synchronization frameworks	Active standard
IEEE 802.1 TSN [77]	Networking/TSN	Enables deterministic Ethernet via time-aware scheduling and traffic shaping	Time-Aware Shaper, Gate Control List, and PTP sync	Active standard
OMG Robotic Interaction Service [78]	Robotics/HRI	A platform-independent framework for HRI data exchange	Platform-independent model for HRI services	Beta specification

AI, artificial intelligence; LLM, large language model; PAR, Project Authorization Request; 3D, 3-dimensional; JSON-RPC, JavaScript Object Notation-Remote Procedure Call; XML, Extensible Markup Language; TEDS, Transducer Electronic Data Sheet; APIs, application programming interfaces; UUIDs, universally unique identifiers; TSN, Time-Sensitive Networking; PTP, Precision Time Protocol; OMG, Object Management Group; HRI, human–robot interaction

While most existing protocols and standards are domain specific, several efforts offer instructive perspectives on standardization across different levels of intelligent systems. At the management level, ISO/IEC 42001:2023 introduces a framework for AI deployment, encompassing organizational accountability, risk oversight, and compliance procedures [34]. For coordination among models and tools, the Model Context Protocol implements a structured client–server schema that supports modular orchestration and dynamic tool invocation in LLM-based systems [35]. At the execution layer, the integration of IEC 61499 and IEC 62541 enables event-driven automation and standardized device-level modeling, forming the backbone of interoperable industrial control [36].

Although these protocols and standards are complementary across different system layers, they remain insufficient to address the coordination challenges posed by embodied tasks. This calls for a closer examination of the practical requirements encountered in the deployment of embodied tasks, which we explore in the following section.

## Requirements

This section derives the core requirements for unified interface protocols in embodied intelligence systems, based on both architectural abstraction and real-world deployment analysis. Building upon the background and coordination challenges discussed in Background and Related Work, it aims to identify recurring patterns that reveal why current systems fail to achieve semantic and operational coherence across perception, planning, and control. Rather than presenting new experimental results, this analysis synthesizes insights from representative deployment pipelines and case studies to distill the underlying coordination requirements that should be addressed by future interface protocols.

### Coordination requirements in typical deployment

**Fig. 2.**
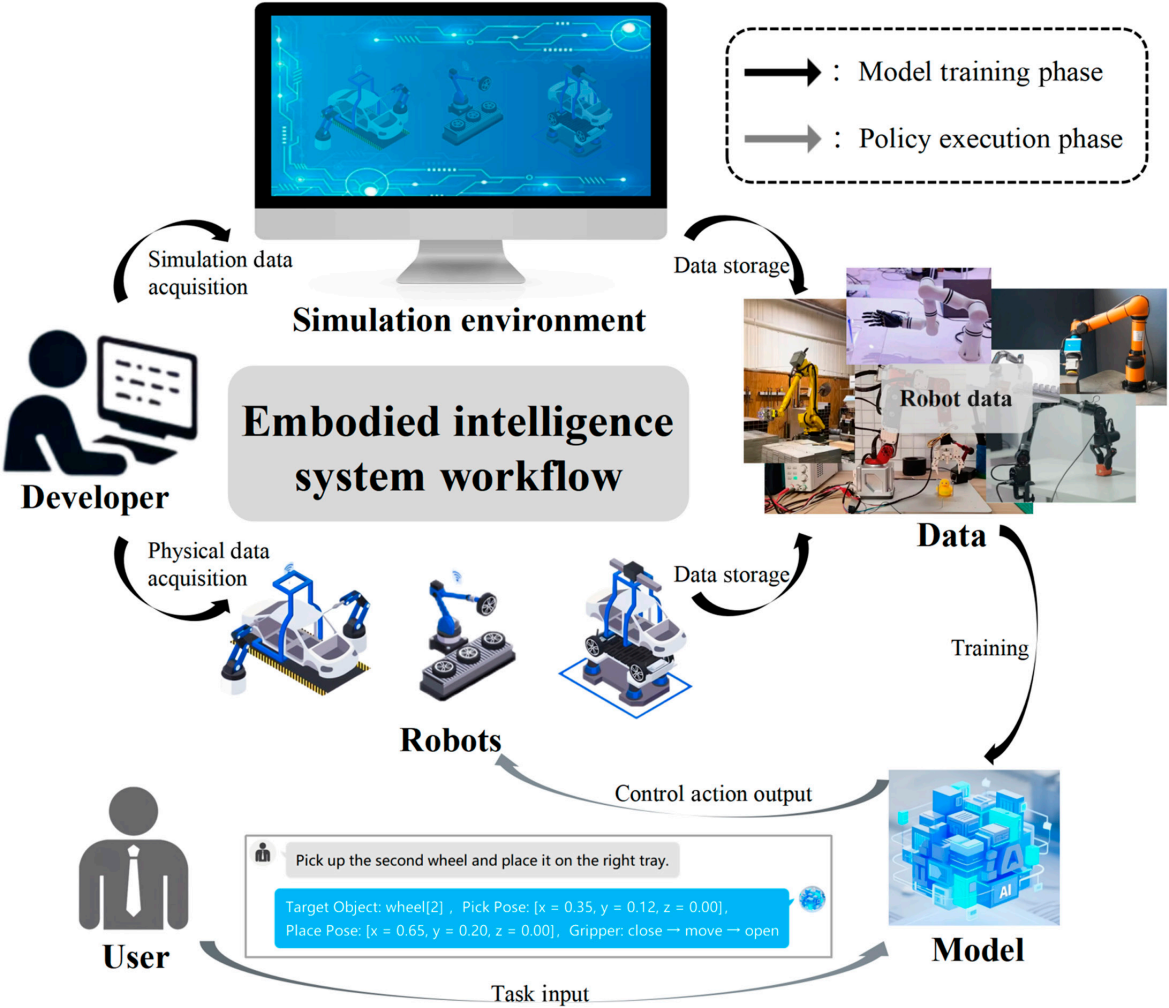
Task workflow of a typical embodied intelligence system, illustrating staged interactions across task definition, data acquisition (physical and simulated), policy model training, and robotic task execution.

Typical embodied deployment, as shown in Fig. 2, involves multiple interacting stages including data acquisition, simulation testing, model training, and task execution, which are typically connected through proprietary interfaces and task-specific integration logic. This structure serves as a representative abstraction of how embodied tasks are typically organized and executed in both academic and industrial settings. However, analyzing this structure through the lens of practical deployment projects exposes persistent coordination issues resulting from incompatible interfaces and ad hoc system composition.

At the data acquisition and training stage, developers routinely face challenges in bridging multisensor environments with model-ready datasets [37]. In systems like Robotics Transformer 1 and Robotics Transformer 2, multimodal inputs including images, joint states, and language instructions are manually formatted into transformer-compatible prompts for vision–language–action (VLA) models [38,39]. These formatting procedures not only are system specific but also lack standard representations of state elements, temporal resolution, or contextual scope [40]. Similarly, simulation-to-real transfer pipelines often require substantial adapter modules to reconcile differences in observation formats, temporal synchronization, and execution feedback between simulated agents and physical robots [41]. In the absence of a structured representation of data semantics and model expectations, incorporating new modalities or models often results in repetitive preprocessing and fragile data alignment procedures.

During execution, most embodied systems suffer from fragmented task coordination. While models may output high-level actions or subgoals, their execution is typically orchestrated through manually defined state machines, imperative control scripts, or task-specific configuration files [42]. In AutoRT, for instance, LLMs interpret user prompts and generate candidate task sequences, which are then routed to specific robots through sampled control policies [43]. However, this execution process lacks a centralized representation of task structure, state progression, or execution feedback, thereby limiting coordinated reasoning across agents. Similarly, Voyager implements a closed-loop agent that relies on prompt chaining and skill routing but provides no abstract interface for task decomposition, failure handling, or cross-skill state persistence [44]. In Xi et al.’s paper [45], LLM-based agents are described as operating with loosely coupled components across memory, perception, reasoning, and action but lack a standardized representation to structure intermodule coordination. These architectural inconsistencies underscore a pressing need for shared task semantics that can support coherent goal decomposition, state tracking, and resource-aware execution in embodied deployments.

Another major disconnect arises between simulated environments and physical robot control systems. While Habitat 2.0 enables rigorous training and evaluation in simulation, deploying the resulting models on physical robots continues to depend on handcrafted interface adaptation and platform-specific control integration [46]. In Isaac Sim, although application programming interfaces (APIs) exist for robot actuation and perception, the underlying state representations differ from those used in ROS-based deployments [47]. Developers are typically required to manually convert simulation outputs into formats compatible with robot controllers by remapping state variables, repackaging sensor data, and synchronizing control interfaces [48]. This lack of consistency in simulation-to-deployment semantics not only increases engineering overhead but also disrupts the continuity of agent behavior and policy evaluation.

These issues reveal a set of recurring coordination requirements that emerge in typical embodied deployments. In the next section, we analyze real-world deployment cases to further uncover deeper requirements.

### Case-driven analysis of requirements

While the previous section abstracted coordination challenges from typical deployment architectures, such structural descriptions only reflect common deployment workflows. In practice, embodied systems reveal more intricate coordination problems across perception, planning, and control when implemented at scale. This section analyzes representative deployment cases to uncover how these issues materialize under real-world constraints and engineering decisions.

Systems powered by LLMs have demonstrated remarkable versatility in translating high-level instructions into structured task plans. In PaLM-E, multimodal inputs comprising visual features, language tokens, and robot states are fused into a unified token stream for downstream reasoning and action prediction [49]. However, the model’s outputs lack standardized representations of intent or execution constraints, requiring downstream components to interpret these outputs using task-specific control logic. CycleIK presents another variant, where symbolic actions generated by Generative Pre-trained Transformer models are chained with open-vocabulary detectors and inverse kinematics modules to realize grasping on humanoid robots [50]. These systems interleave reasoning and execution, but coordination logic remains fragmented and task structure is often hardcoded [51]. In Gan et al.’s study [52], a bionic robot controller is developed for dual-arm collaboration, combining perception, planning, and motion execution via ROS and industrial systems. Although the system uses modular components, coordination across tasks and arms depends on tightly coupled logic and handcrafted message passing. In Fan et al.’s paper [53], LLMs are used to interpret task descriptions, generate toolpaths, and execute actions via modular prompts and G-code templates. However, the execution logic remains hardwired, with no standardized abstractions for task memory or cross-module coordination. As a result, model outputs must be interpreted through system-specific control pipelines, making integration brittle and difficult to generalize.

Multimodal embodied systems that integrate language, vision, and control often rely on ad hoc structures to inject task semantics and contextual information into their execution pipelines. In GR-MG, for instance, a diffusion-based generator produces goal images conditioned on language instructions and task progress, which are then paired with partially annotated demonstrations to predict action sequences [54]. However, progress tracking is not represented as a formal interface, and task semantics must be reinferred at runtime, leading to redundancy across components. In Mon-Williams et al.’s study [55], a different strategy is adopted, where LLMs are used to generate Python control code from prompts, supplemented by memory modules and external code references. Yet the system lacks modular abstraction: error handling, knowledge retrieval, and execution control are tightly coupled in task-specific scripts. Scene-driven methods such as Scene-MMKG and its instantiated variant ManipMob-MMKG construct multimodal knowledge graphs that encode perceptual and apperceptive information for embodied agents [56]. While these knowledge bases provide enriched semantic context, their use in downstream pipelines still relies on manually designed schemas, similarity-based queries, and scene-specific alignment heuristics, which ultimately hinders generalization across tasks.

Deploying shared policy models across different robot systems often exposes concrete coordination barriers in action binding, control adaptation, and interface alignment [40,51]. In O’Neill et al.’s paper [57], shared policy models are trained across a wide variety of robot morphologies using aligned datasets, yet deploying those models still requires tailoring action schemas and bindings for each robot’s control stack. A similar issue arises in RoboMIND, where policy evaluation spans multiple robot embodiments, but task execution still depends on platform-specific definitions and manually structured skill sequences [58]. In the DEXBOT framework, which integrates embodied AI modules for high-precision construction tasks, coordination remains a challenge [59]. Despite its task decomposition pipeline and modular learning architecture, the system relies on manually configured transitions between perception, physical modeling, and control stages, limiting reusability and increasing integration overhead across different deployment scenarios. In Wang et al.’s paper [32], embodied foundation models operate across diverse physical and virtual agents, yet the system still depends on fragmented memory handling and task-specific execution logic, which limits consistency across agents and increases the burden of system adaptation. These cases illustrate how even when high-level policies can be shared across embodiments, deployment often breaks down at the level of action mappings, hardware abstraction, and system configuration, resulting in reliance on handcrafted logic and expert-driven tuning.

For clarity, Table 2 presents a categorized slate of requirements (R1 to R14). Through the analysis of deployment requirements in these cases, it becomes evident that even in leading-edge architectures, coordination is rarely treated as a first-class concern. This reflects a structural blind spot in the design of embodied deployments, one that calls for a unified interface construct to govern task-, model-, and control-level interactions in a system-agnostic and extensible way.

**Table 2.** Embodied system coordination requirements distilled from architectural abstraction and real-world deployment evidence

ID	Requirement	Sources	Category	Layer
R1	Typed observation and action semantics with explicit model input–output contracts covering shapes, units, frames, timing, and context window	[38–40]	Semantics and input–output	Semantic
R2	Extensible modality schema with versioned fields and declared context scope to accommodate new sensors and models without bespoke preprocessing	[37,40]		
R3	Portable representation of the task structure with a uniform progress and failure envelope for orchestration and recovery	[42,43,45]	Orchestration and workflow	Workflow
R4	Workflow transition contracts that formalize handoffs between perception, physical modeling, and control stages	[52,59]		
R5	Interface-level normalization of units, frames, and clocks to secure observational parity between simulation and physical robots	[41,46,47]	Simulation to real and portability	Adapter
R6	Machine-readable capability manifests and portable binding profiles for actions, resources, and controllers to support cross-embodiment deployment	[57,58]		
R7	Evaluation and benchmarking parity across simulation and hardware, ensuring comparable datasets, metrics, and interface semantics	[46,47]		
R8	Standardized intent representation with preconditions, postconditions, and execution constraints linked to executable skills	[49,50,53]	Intent and skills	Interaction
R9	First-class primitives for progress tracking and episodic provenance that enable faithful replay and auditing	[32,54]	State and provenance	Interaction
R10	Shared runtime context for cross-module and cross-skill state persistence rather than pipeline-specific memory	[32,44]		
R11	Dataset construction and preprocessing provenance aligned with policy expectations to reduce mismatch at training and deployment	[38]		
R12	Unified execution feedback and telemetry schema with structured status, timing, and error information across modules and devices	[48,55]	Telemetry, timing, and recovery	Interaction
R13	Declared time base, rate, and latency budgets with synchronization semantics for coordinated scheduling	[41,48]		
R14	Standard error taxonomy and recovery strategies with observable states for diagnosis and automated fallback	[42,43]		

## Overview of ECP

Building upon the coordination requirements identified in the previous section, this section introduces ECP—a unified interface specification designed to address interoperability and semantic alignment challenges in embodied intelligence systems. The following subsections present its design philosophy, interface specification, execution workflow, and current progress.

### Design philosophy and positioning

#### Design principles

The design of ECP is guided by several foundational principles that define its architecture and intended functionality.

• Task-oriented interoperability: ECP is intended to support coordination across diverse modules by introducing a consistent and structured interface format. The protocol enables motion planners, inference models, robot actuators, and perception units to be scheduled and interpreted based on task roles, rather than purely on message routing or data format matching.
• Modular abstraction with executable semantics: Each interface element in ECP encapsulates a meaningful unit of execution, structured with input–output signatures, preconditions, model bindings, and execution state indicators. This design allows developers to compose, trace, and adapt task workflows at the semantic level.
• Protocol-level extensibility: ECP defines a series of versioned core schemas that can be extended via formally typed optional fields and namespaced types to introduce domain-specific constructs, ensuring long-term adaptability to new hardware modules, reasoning frameworks, and task logics while preserving backward compatibility.
• Alignment between simulation and physical systems: One of the key design considerations in ECP is maintaining consistent interfaces across simulation and deployment environments. By unifying the data structures and semantic descriptions used for training and real-world execution, the protocol helps reduce the cost and complexity of sim-to-real transfer.
• Declarative coordination and introspection: ECP promotes declarative orchestration via well-typed workflows that compose interaction verbs over semantic resources. Uniform progress semantics and discovery manifests enable principled supervision, failure handling, and portability across backends.

#### Objectives

The specific goals of ECP can be summarized as follows:

• Enable AI models, robotic systems, and digital tools to interact through a standardized, task-aware interface.
• Support structured execution workflows that reflect task hierarchies and model dependencies.
• Bridge the gap between simulation and deployment by enforcing consistency in interfaces and data semantics.
• Reduce the complexity of module integration in embodied systems through clear and reusable interface definitions.
• Provide a foundation for building scalable, interoperable, and adaptive embodied intelligence architectures.

These principles and objectives position ECP as a mid-level semantic interface specification and declarative composition model for managing execution context, aligning interface semantics, and supporting modular coordination in embodied systems.

### Interface specification

Based on the design principles outlined above, ECP is an interface protocol that adopts a layered architecture to formalize how semantics, interactions, and workflow compositions are represented and executed across embodied systems. The protocol standardizes semantic and coordination interfaces, enabling heterogeneous modules such as models, simulators, and controllers to communicate and coordinate within a unified context description. Guided by the requirements distilled in Table 2, ECP is divided into 4 protocol layers: the Semantic Layer, Interaction Layer, Adapter Layer, and Workflow Layer.

#### Semantic Layer

Addressing the semantics and input–output requirements (R1 and R2) in Table 2, the Semantic Layer specifies transport-agnostic schemas for observations, actions, and task context used throughout embodied workflows. The schemas make temporal resolution, unit system, and spatial frame explicit, together with versioning and context fields. Making these descriptors explicit reduces semantic drift between perception and control stacks, supports ordering and latency analysis, and allows producers and consumers to evolve without breaking compatibility. In practice, the same type definitions can be bound to ROS topics, simulator buffers, or archival backends without redefinition, improving reproducibility and easing sim-to-real alignment. Concretely, the header carries nanosecond-resolution time, unit system, spatial frame, version, and context. At interface boundaries, we perform consistency checks to ensure that the per-stream time is monotonic and that unit and frame conversions are explicit.

#### Interaction Layer

The Interaction Layer primarily addresses the requirements on intent and skills (R8), state and provenance (R9 to R11), and telemetry, timing, and recovery (R12 to R14) in Table 2. It provides a stack-independent invocation contract: interactions use a compact verb vocabulary $\begin{array}{c} V=\left\{\text{read},\text{write},\text{execute},\right. \end{array}$$\begin{array}{c} \left.\text{subscribe},\text{append},\text{query},\text{discover}\right\} \end{array}$ over path-addressable semantic resources, and progress is modeled by a finite state $s\in \left\{\text{accepted},\text{in}\_\text{progress},\text{completed},\text{failed}\right\}$. Response envelopes include structured status and timing information, while requests declare time-outs and deadlines, as well as budgets for rate and latency, making supervision and recovery consistent across backends. Each operation is linked to shared runtime context and provenance, which preserves cross-module state and enables faithful replay and auditing. In practice, the layer brings sensing, actuation, and inference under the same coordination semantics and simplifies workflow composition across heterogeneous systems.

#### Adapter Layer

The Adapter Layer operationalizes the simulation to real comparability and portability highlighted in Table 2 (R5 to R7). It binds the protocol’s semantic and interaction abstractions to backend APIs, including ROS 2 interfaces, programmable logic controller (PLC) runtime APIs, OPC UA server endpoints, simulators, and archival backends, while preserving the protocol’s semantics. Following Asset Administration Shell-based information modeling for manufacturing systems [60], the Adapter Layer uses unit-annotated, semantically referenced properties with administrative timestamps. Building on this basis, it explicitly exposes units, frames, and time bases at the interface boundary and performs pre-invocation schema and capability checks. By clarifying these boundary rules, a single workflow specification can target simulation and physical systems with consistent interpretation, results remain comparable across heterogeneous backends, and rebinding effort is reduced. In practice, this layer enables portable deployment and evaluation while preserving protocol semantics across diverse systems.

#### Workflow Layer

The Workflow Layer primarily addresses the orchestration and workflow requirements (R3 and R4) in Table 2. It provides a declarative composition framework that connects the verbs of the Interaction Layer with the objects of the Semantic Layer to form structured task sequences. Each workflow represents a context-aware trace of execution, where inputs, outputs, and dependencies are explicitly defined and can be statically verified. Drawing on the function block orchestration style of IEC 61499 [61], this layer represents embodied tasks, including data acquisition, policy training, and physical execution, as explicit graph structures that support portable deployment and auditable process representation. Execution progress, failure semantics, and retry policies are uniformly defined, ensuring that workflows remain reproducible and interpretable across both simulated and physical settings.

Collectively, these 4 layers provide a unified framework for expressing and managing the life cycle of embodied intelligence tasks from data acquisition and model training to inference and control. The layered architecture not only reduces the integration burden between heterogeneous systems but also introduces a formal mechanism to maintain semantic consistency across diverse operational contexts.

### ECP workflows in system architectures

This section illustrates how ECP manifests within real-world embodied system workflows and demonstrates its role in mediating coordination among heterogeneous functional modules. Rather than describing a single implementation, the discussion generalizes from a representative deployment architecture developed in our laboratory to exemplify how ECP interfaces structure task-level interactions across practical embodied applications.

**Fig. 3.**
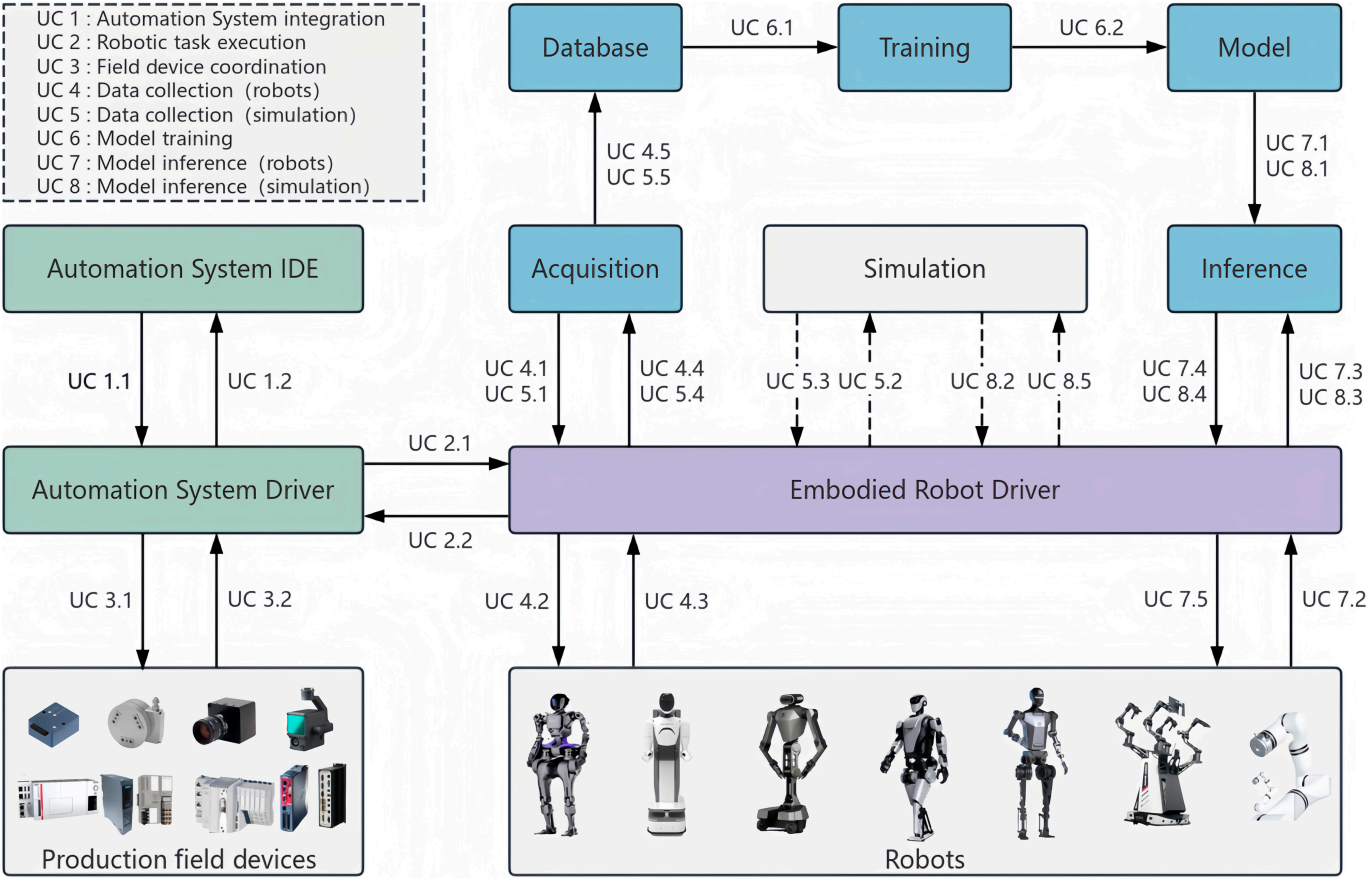
This architecture of an embodied intelligence system illustrates how the Embodied Context Protocol (ECP) is embedded within practical task workflows by mediating coordination among functional modules. The architecture is derived from an actual deployment scenario developed in our laboratory. IDE, integrated development environment.

As shown in Fig. 3, the architecture encapsulates the primary components of an embodied intelligence system, including task modeling and orchestration (Automation System integrated development environment [IDE]), control translation (Automation System Driver), model management (Model Module), and auxiliary services for data acquisition, simulation, and training. These components are interconnected through standardized ECP interfaces that formalize information flow, task context, and control semantics across stages of embodied task execution.

To describe how coordination unfolds across the architecture, a set of 8 representative interaction pathways (UC 1 to UC 8) are defined, each capturing a distinct stage of the embodied task life cycle—from task configuration and model invocation to physical execution and feedback collection. For instance, UC 4 corresponds to the robot-driven acquisition of multimodal sensory data, while UC 6 concerns the retrieval and utilization of archived task data for model training. Each use case exemplifies a recurring coordination pattern in embodied workflows, expressed through ECP verbs and semantic resources.

Taking UC 4 as an example, a structured acquisition request is issued from the Data Acquisition Module to the Robot Driver, specifying sensor modalities, sampling frequency, and synchronization policy. ECP encapsulates this interaction as a standardized schema containing contextual and temporal descriptors, enabling consistent interpretation across backends. Subsequent subflows such as UC 4.4 govern the preprocessing and temporal alignment of multimodal data, ensuring reusability in training pipelines. Beyond data acquisition, analogous coordination patterns appear in inference, simulation, and task dispatch, each represented as declarative ECP workflows rather than procedural scripts.

Within this layered architecture, ECP interfaces act as semantic contracts that unify interactions across perception, reasoning, and control subsystems. By standardizing context representation, progress semantics, and discovery manifests, they enable transparent supervision and recovery throughout task execution. This system-level abstraction demonstrates how ECP operationalizes the coordination principles introduced earlier, bridging symbolic orchestration and low-level actuation in both simulated and physical environments. The following section presents a concrete validation case based on a humanoid picking-and-placing task.

### Work in progress

**Fig. 4.**
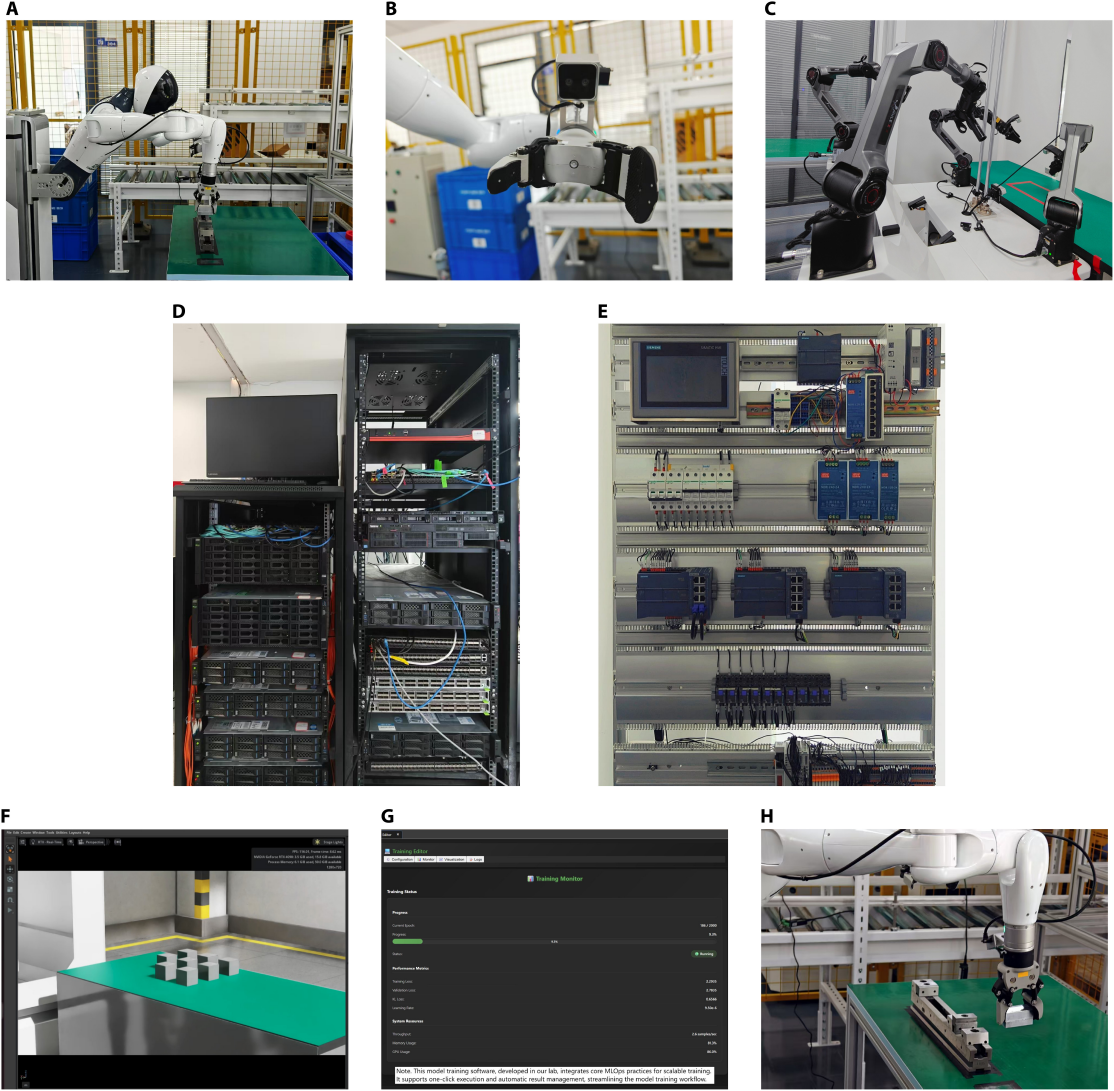
Real-world deployment scenarios of embodied task workflows, including (A and B) a humanoid robotic system performing multiview perception and picking operations (type: AgiBot G1), (C) a dual-arm platform (type: AgileX Robotics COBOT Magic), (D) local fog servers hosting the Action Chunking Transformer (ACT)-based policy model, (E) an industrial control cabinet integrating programmable logic controllers (PLCs) and sensor interfaces to decompose high-level tasks into atomic operations, (F) the data acquisition interface in the simulation environment, (G) the model training interface developed in our lab, and (H) model inference executed on the humanoid robot.

This subsection reports an engineering implementation that illustrates how ECP interfaces are instantiated across perception, inference, and control in a real deployment. This implementation vignette is descriptive and noncomparative; it does not present controlled baselines, statistical tests, or quantitative claims. In this case, ECP was applied to a pick-and-place task performed by a humanoid robot, comprising a sequence of operations including visual guidance, object localization, and policy-driven manipulation. As illustrated in Fig. 4, the deployment scenario comprises 3 primary components: a humanoid robot equipped with head-mounted and arm-mounted cameras (Fig. 4A), local fog servers running an Action Chunking Transformer (ACT)-based policy inference service (Fig. 4D), and a PLC-based control subsystem responsible for real-time actuation and task decomposition (Fig. 4E). These components are connected through ECP interfaces that ensure uniform data semantics and task context propagation along the workflow, from sensory acquisition to model inference and physical execution.

Before initiating the task, an ACT-based policy model was constructed and deployed to support downstream decision-making. The process began with multimodal data acquisition in a simulated environment of robotic interactions (Fig. 4F), where synchronized visual, proprioceptive, and control signals were collected to build a task-specific dataset. These data were then used to train the policy model (Fig. 4G), ensuring that the input and output formats followed the unified semantic schema defined by ECP. After training, the model was deployed onto the physical robot for on-robot inference (Fig. 4H), with the same interface structures preserving compatibility between simulation and real-world execution. With the model infrastructure in place, the picking workflow unfolded in 2 main stages, each involving distinct sensory and control pathways. In the first stage, when the target object was clearly visible in the robot’s first-person view, the head-mounted camera provided visual feedback for a primary picking attempt (Fig. 4A). When the object was partially occluded or outside the immediate field of view, a fallback mechanism activated the arm-mounted camera to obtain supplementary perception (Fig. 4B), which was processed through the same standardized observation interface. If the initial pick was inaccurate or required refinement, the system entered a second stage of secondary localization and actuation, during which the ACT model was invoked to handle complex object poses and refine the grasp trajectory. Based on updated positional estimates and feedback from the control layer, the picking motion was adaptively refined to ensure accurate and robust execution. Upon completing the pick, the placing phase was executed under visual and positional supervision, ensuring proper alignment and secure placement at the designated target. Throughout this entire process, ECP served as the coordination interface connecting perception, inference, and control components, maintaining uniform data semantics, consistent task context, and traceable execution progress across heterogeneous modules.

To better support embodied tasks, we expose a suite of lab-developed auxiliary tools as ECP endpoints. Representative examples include Bag2HDF5, which converts ROS 2 rosbag recordings into schema-aligned HDF5 episodes, and V-Player, a multicamera feature extraction and visualization service that subscribes to multiple image channels and model-side feature taps during both training and inference. These tools integrate through standard ECP interfaces and report via the unified progress semantics, providing enhanced transparency and traceability throughout data and model workflows.

Complementing the humanoid deployment, we also conducted data collection and evaluation on the AgileX Robotics COBOT Magic dual-arm platform (Fig. 4C), including validation with ACT and VLA models. This effort yielded an industrial manipulation dataset of 2,320 multimodal episodes [62]; a complementary study systematically assesses the transfer and performance of VLA models under industrial disturbances [63].

Across the stages of the picking-and-placing task, the following qualitative engineering observations were recorded for the ECP-based deployment:

• In earlier versions of the task, manual adjustment of data formats and custom coordination scripts was often required to integrate different modules. With the introduction of ECP interfaces, connections between sensing, decision, and execution components were standardized, enabling standardized data exchange across stages.
• By connecting the Automation System IDE and the ACT model through ECP interfaces, the original long-horizon workflow was decomposed into shorter, clearly defined execution phases. This modularization enabled precise execution tracking and stage-level recovery in complex picking tasks.
• The use of typed ECP interfaces to connect models with training and deployment software simplified reuse of collected data for policy fine-tuning and enabled predefined recovery steps in execution flows. Together, these mechanisms facilitated iteration in practice and supported safety-oriented recovery.
• By exposing a suite of tools as endpoints via ECP interfaces, the in-loop availability and diagnostic capability of the tools were increased, enabling better observability and short debugging cycles. For example, Bag2HDF5 reduced format-handling overhead and strengthened dataset consistency, while V-Player provided runtime visibility and diagnosability.

The successful application and validation of ECP within our current humanoid robot picking-and-placing scenario clearly demonstrate the protocol’s practical effectiveness and maturity for real-world engineering deployment. By addressing integration challenges, enhancing execution safety, and accelerating both system deployment and policy model iteration, these results confirm the viability of ECP as a foundational interface protocol for future embodied intelligence systems. Moreover, the demonstrated capabilities lay the groundwork for broader industry adoption, highlighting the potential of ECP to streamline cross-domain deployments and support scalable, maintainable, and robust embodied intelligence applications.

## Future Directions and Challenges

Grounded in the demonstrated results, this section examines how ECP is structurally positioned to scale, adapt, and integrate across broader embodied intelligence applications while also outlining unresolved challenges that will shape its standardization and adoption.

### Semantic interoperability across domains

As embodied intelligence systems continue to integrate diverse sensing modalities, learning models, and execution backends, ensuring interoperability at the level of semantic intent becomes essential [9]. This includes not only compatibility in data formats but also agreement on task roles, capability constraints, and execution context.

To support this, ECP interfaces are specified not only by structural types but also by task-relevant ontologies that encode the intended function, outcome semantics, and applicable conditions of each interaction. For instance, an interface for tool handover may describe not only motion parameters but also role assignments, safety constraints, and timing dependencies. These domain-aligned schemas provide a shared vocabulary for modules to reason about each other’s capabilities and responsibilities. By incorporating lightweight semantic annotations, such as those expressed through task graphs or ontology-linked descriptors, ECP allows reasoning engines to perform compatibility checks and substitute components in open-ended execution settings.

Semantic interoperability in embodied workflows also requires bridging across modalities. A typical embodied task may involve visual inputs, language prompts, force feedback, and symbolic planning [53]. ECP provides a unifying interface abstraction that allows these multimodal signals to be jointly expressed and interpreted. For example, a policy module may receive a structured ECP context object that includes a segmented image, a parsed instruction, and a robot state vector. By supporting such fused representations within the protocol schema, ECP enables more robust and generalizable interaction pipelines across sensory and cognitive boundaries.

Beyond single-agent systems, ECP is also designed to enable semantic coordination in distributed multiagent environments. In such settings, agents must negotiate shared task structures, synchronize capabilities, and maintain state consistency despite differences in embodiment or computation. ECP supports the structured expression of semantics relevant to coordination, including capabilities, execution states, and shared task context, enabling the coordination layer to handle execution anomalies through a standardized interface substrate. By providing a shared coordination contract, ECP facilitates modular alignment and collaborative planning, allowing heterogeneous agents to interact through common interface semantics rather than hardwired behavior links.

More broadly, although our illustrations center on industrial deployments, future work will extend ECP to other domains, such as healthcare and service robotics, by reusing the protocol’s invariant semantic objects, interaction verbs, and workflow semantics, together with domain-specific adapters and safety profiles.

### Scalable interface ecosystem

The conception of ECP is grounded in the understanding that embodied intelligence systems are progressing toward increasing complexity, modularity, and deployment scale. Rather than targeting fixed pipelines or isolated scenarios, ECP is positioned as a scalable and composable interface protocol that supports the integration of heterogeneous modules across varied operational contexts while ensuring semantic coherence and consistent task coordination.

At the core of this vision is the abstraction of system interactions into semantic interfaces that are independent of specific implementation details. ECP defines interfaces not as static function calls but as context-aware contracts that specify task roles, parameter expectations, and execution semantics. This level of abstraction enables systems to incorporate new modules and workflows without altering existing coordination logic.

To operationalize this abstraction in real-world environments, ECP introduces a mechanism for capability declaration. Each module can describe its functional offerings using structured profiles that define supported interface types, input and output specifications, and execution constraints. These capability descriptions enable orchestration layers to construct workflows based on functional compatibility and task requirements, rather than relying on predefined or hardcoded bindings. At scale, compatibility across heterogeneous hardware is handled by declaring device-class capability profiles and making unit, spatial frame, and clock normalization explicit, which yields portable bindings across robots and controllers.

In addition to modular matching, ECP is also designed to support hierarchical composition of task workflows. Complex behaviors can be decomposed into smaller sub-interactions, each associated with a distinct interface. For example, adaptive picking in partially occluded environments may involve a sequence of operations such as visual segmentation, multiview reconstruction, contact-aware control, and motion adjustment. Each step can be represented through a dedicated interface instance within a nested execution graph. This composition model improves modular reuse, enhances clarity in task logic, and supports transparent execution tracking.

To promote extensibility across diverse application domains, ECP will provide a formal registration mechanism for interface schemas. Developers and research communities can define and publish new interface types and capability templates based on domain-specific needs. This mechanism enables the introduction of specialized schemas, such as those for vision and language grounding, multiagent coordination, or safety-critical robotic behavior, without modifying the protocol core. By decoupling extension from implementation, ECP supports protocol evolution while preserving coherence and interoperability across systems. Practically, the registry enables conformance tests, compatibility matrices, and declared downgrade paths. When workflows are composed with stage boundaries and checkpoints, they support back pressure and bounded retries, which improves system-level scalability without changing the protocol core.

### Standardization and system integration

A central goal of ECP is to support seamless coordination across heterogeneous components, ranging from high-level AI modules to low-level industrial devices. To achieve this, ECP is designed as a semantic coordination layer that complements, rather than replaces, existing communication and automation standards. Its semantic structure and modular abstraction allow it to interoperate with widely adopted protocols while providing the missing task-level coordination semantics required by embodied intelligence systems.

ECP is particularly compatible with industrial standards such as OPC UA, IEC 61499, and Data Distribution Service, which govern device communication, event-driven logic, and distributed messaging. These protocols address system integration at the signal and control levels but typically lack abstractions for task semantics, model invocation, or context-aware behavior planning. ECP bridges this gap by providing an upper-layer coordination contract that binds task logic to executable behavior across physical and simulated systems. For example, a planning module that uses ECP interfaces can generate goal-oriented instructions that are translated into OPC UA-compliant commands, preserving semantic integrity throughout the execution chain.

In parallel, ECP is structured to serve as an interface definition mechanism for development toolchains. It can be used to define, validate, and deploy workflows across simulation engines, robotic IDEs, and deployment frameworks. By capturing interface semantics in a machine-readable format, ECP enables automatic workflow synthesis, version-controlled interface management, and runtime reconfiguration of modules. For intelligent systems that rely on evolving policy models, ECP further supports standardized model invocation contracts, allowing decoupled deployment and dynamic runtime binding without altering upstream orchestration logic.

By supporting integration at both the protocol and toolchain levels, ECP provides a pathway toward unifying AI reasoning, symbolic planning, real-time control, and physical actuation. Its compatibility with existing standards and modular deployment environments positions it as a practical and scalable interface layer for the next generation of embodied intelligence systems.

### Unresolved challenges

While the preceding sections highlight concrete future directions, the following unresolved challenges will shape the standardization and large-scale adoption of ECP. The first challenge lies in achieving finer-grained semantic abstractions while at the same time meeting real-time guarantees, which is essential for heterogeneous and multiagent deployments. Another critical issue concerns cross-platform compatibility across diverse robots, sensors, models, and simulators, which will require portable profiles and standardized adapters to ensure consistent behavior. Equally important is the alignment with safety-critical and time-sensitive domains, demanding integration with established standards as well as robust mechanisms for runtime assurance. Ultimately, the creation of a sustainable ecosystem will depend on reproducible benchmarks, certified conformance, and tools that enable modular reuse at scale.

Collectively, these future directions and unresolved challenges define the frontier of embodied intelligence interface research. Their resolution will require not only technical advances in semantic design, real-time guarantees, and cross-platform tooling but also collective effort toward reproducible benchmarks, certified conformance, and community-driven standards. Concretely, ECP uses semantic versioning with deprecation windows and capability negotiation to preserve backward compatibility. We maintain companion profiles and reference adapters, and we are pursuing a formal standardization path with the International Organization for Standardization/International Electrotechnical Commission and Institute of Electrical and Electronics Engineers, with a near-term road map of a schema registry, validators, and conformance tests. Addressing them will determine whether ECP can evolve from an early stage protocol into a protocol standard that unifies laboratory innovation with industrial deployment at scale.

## Conclusion

The development of embodied intelligence has entered a stage where integration, rather than perception or control alone, defines the frontier of progress. This review has traced the structural challenges that emerge when perception, reasoning, and actuation are distributed across heterogeneous modules, revealing that the absence of a unified task-level interface has become the key barrier to scalable and reproducible embodied deployments. From this foundation, ECP is proposed and analyzed as an emerging solution that formalizes the semantic and operational coherence required for large-scale coordination.

Building on deployment pipelines and case analyses, recurring coordination requirements emerge: context semantics, capability declaration, workflow composition, and sim-to-real consistency. ECP addresses these challenges by providing a layered interface specification. Looking ahead, advancing ECP from protocol specification to ecosystem adoption will require progress in standardization, benchmarking, and cross-domain integration. Ongoing collaboration between academia, industry, and standardization bodies will be pivotal in advancing ECP’s adoption, positioning it as a foundational protocol for the next generation of embodied intelligence systems.

## Data Availability

No new data were generated or analyzed in this study.
